# Supplementary material for: Upregulated Expression of Activation-Induced Cytidine Deaminase in Ocular Adnexal Marginal Zone Lymphoma with IgG4-Positive Cells
Source: Int J Mol Sci. 2021 Apr 15;22(8):4083. doi: 10.3390/ijms22084083 (PMC8071226; doi:10.3390/ijms22084083)
Supplement: Supplementary file 1 [file ijms-22-04083-s001.zip › ijms-1157465-supplementary.pdf]

No.	Diagnosis	Sex	Age	IgG4+ cells (/hpf)	IgG+ cells (/hpf)	IgG4+/IgG+ cell ratio	Serum IgG4 (mg/dL)	Serum IgG (mg/dL)	Serum IgG4/IgG
1	IgG4-ROD	M	61	184	355	0.518	197	1536	0.128
2	IgG4-ROD	M	75	126	160	0.788	—	—	—
3	IgG4-ROD	F	65	160	301	0.532	268	1465	0.183
4	IgG4-ROD	M	77	365	409	0.892	969	2454	0.395
5	IgG4-ROD	F	66	260	365	0.712	106	1400	0.076
6	IgG4-ROD	M	60	178	309	0.576	—	293.2	—
7	IgG4-ROD	M	73	137	198	0.692	136	1437	0.095
8	IgG4-ROD	F	56	177	275	0.644	—	1748	—
9	IgG4-ROD	M	49	270	323	0.836	515	1659	0.31
10	IgG4-ROD	F	45	127	142	0.890	97.4	1518	0.064
11	IgG4-ROD	F	60	184	223	0.825	120	1499	0.08
12	IgG4-ROD	M	72	311	474	0.656	—	—	—
13	IgG4-ROD	F	58	207	248	0.835	376	1351	0.278
14	IgG4-ROD	M	61	298	440	0.677	3320	4168	0.797
15	IgG4-ROD	F	36	160	174	0.920	561	1958	0.287
16	IgG4-ROD	M	67	85	123	0.691	377.1	1369.4	0.275
17	IgG4- MZL	M	72	49	164	0.299	—	—	—
18	IgG4- MZL	F	39	2	82	0.024	194	1539	0.126
19	IgG4- MZL	F	56	16	108	0.148	—	—	—
20	IgG4- MZL	M	69	44	259	0.170	—	1407	—
21	IgG4- MZL	F	74	1	12	0.083	—	1279.4	—
22	IgG4- MZL	M	74	0	12	0.000	—	—	—
23	IgG4- MZL	M	69	47	669	0.070	—	—	—

24	IgG4- MZL	F	66	0	15	0.000	19.3	962	0.02
25	IgG4- MZL	F	71	25	605	0.041	—	1844	—
26	IgG4- MZL	M	61	0	4	0.000	19.8	1092	0.018
27	IgG4- MZL	M	82	0	26	0.000	166	1520	0.109
28	IgG4- MZL	M	39	2	17	0.118	—	—	—
29	IgG4+ MZL	F	82	133	102	1.304	226	1356	0.167
30	IgG4+ MZL	M	41	355	240	1.479	1090	1506	0.724
31	IgG4+ MZL	M	64	223	230	0.970	—	—	—
32	IgG4+ MZL	F	57	104	251	0.414	—	—	—
33	IgG4+ MZL	F	42	123	152	0.809	226	1356	0.167
34	IgG4+ MZL	M	72	128	152	0.842	760	2709	0.281
35	IgG4+ MZL	F	65	143	300	0.477	116	1450	0.08
36	IgG4+ MZL	F	57	128	183	0.699	144	1480	0.097
37	IgG4+ MZL	F	71	115	283	0.405	450	1844	0.244
38	IgG4+ MZL	M	56	191	205	0.932	475	1536	0.309
39	IgG4+ MZL	M	60	137	113	1.206	230	1458	0.158
